# Factors associated with reflux resolution in extravesical laparoscopic and robotic surgery

**DOI:** 10.1002/bco2.70054

**Published:** 2025-07-22

**Authors:** Kentaro Mizuno, Hidenori Nishio, Daisuke Matsumoto, Takuya Sakata, Akihiro Nakane, Hideyuki Kamisawa, Satoshi Kurokawa, Tetsuji Maruyama, Keiichi Tozawa, Takahiro Yasui, Yutaro Hayashi

**Affiliations:** ^1^ Department of Pediatric Urology Nagoya City University Graduate School of Medical Sciences Nagoya Japan; ^2^ Department of Nephro‐urology Nagoya City University Graduate School of Medical Sciences Nagoya Japan; ^3^ Department of Urology Nagoya City University West Medical Center Nagoya Japan; ^4^ Department of Urology Gamagori City Hospital Gamagori Japan; ^5^ Department of Urology Anjo Kosei Hospital Anjo Japan; ^6^ Department of Urology Nagoya City University East Medical Center Nagoya Japan; ^7^ Department of Medical Safety Management Nagoya City University Graduate School of Medical Sciences Nagoya Japan

**Keywords:** extravesical, laparoscopy, robotic surgical procedures, urinary retention, vesicoureteral reflux

## Abstract

**Objective:**

This study aims to assess the surgical outcomes of laparoscopic or robotic surgery for primary vesicoureteral reflux and elucidate the factors contributing to vesicoureteral reflux resolution.

**Patients and Methods:**

We retrospectively reviewed the medical records of consecutive patients who underwent extravesical laparoscopic ureteral reimplantation or robot‐assisted laparoscopic ureteral reimplantation at our institution between March 2012 and July 2020. First, we reviewed surgical outcomes in the paediatric (*n* = 100) and adult (*n* = 15) patient groups. Second, we compared the surgical findings and outcomes of both procedures in the paediatric patient group and investigated the factors contributing to surgical success in the paediatric patient group.

**Results:**

The combined success rates for both procedures were 89.1% in the paediatric group and 70.0% in the adult group. The overall success rate among paediatric patients was not significantly different between those who underwent laparoscopic ureteral reimplantation (91.1%) and those who underwent robot‐assisted laparoscopic ureteral reimplantation (85.5%). Post‐operative urinary retention was observed in 5.0% and 6.7% of paediatric and adult patients, respectively. Univariate and multivariate analyses revealed that the ureteral diameter measured during surgery was significantly associated with vesicoureteral reflux resolution regardless of the use of a robotic platform (*p* = 0.046).

**Conclusion:**

Both laparoscopic and robot‐assisted laparoscopic ureteral reimplantation are favourable and safe procedures for paediatric patients with primary vesicoureteral reflux. To improve the success rate, a sufficient length of the submucosal tunnel must be ensured based on intraoperative measurements of ureteral diameter.

## 1 INTRODUCTION

Vesicoureteral reflux (VUR) varies according to age, sex, voiding function, grade and comorbidities; the best treatment procedure for it remains debatable.^1^ Although there has been a steady decline in VUR procedures in the United States^2^ and Finland,^3^ open surgeries have been replaced with minimally invasive surgery (MIS). With the recent progress in MIS, laparoscopic and robot‐assisted methods are gaining popularity.^4^ According to a recent systematic review,^5^ the MIS approach results in shorter hospital stay, decreased blood loss and decreased wound infection. Moreover, MIS is equivalent to open surgery in terms of success rate (SR) and post‐operative complications. In particular, extravesical approaches have become primary procedures because of trocar placement issues and challenges in manoeuvring the instruments within small‐capacity bladders via an intravesical approach.^6^ Recently, robot‐assisted laparoscopic ureteral reimplantation (RALUR) has been widely used in paediatric patients,^7^ resulting in improved surgical outcomes.^8^ In addition, RALUR is performed in patients with high‐grade VUR,^9^, ^10^ after injection therapy, or in patients with megaureter,^11^ suggesting that the surgical indications for RALUR are increasing. To stabilize and improve the surgical outcomes of RALUR compared to that of open surgery for VUR, surgical or patient factors associated with RALUR need to be elucidated. Gundeti et al. have advocated for the ‘LUAA’ technique, stressing the length of the submucosal detrusor tunnel (L), use of a U stitch (U), a permanent apical stay suture (A) and incorporation of the ureteral adventitia (A) during detrusorraphy.^12^ However, a comprehensive assessment of predictive factors for surgical success in patients who undergo MIS for VUR remains unclear. In this study, we aimed to retrospectively assess the surgical outcomes of patients who underwent laparoscopic ureteral reimplantation (LUR) or RALUR and to elucidate the factors contributing to the resolution of VUR.

## 2 PATIENTS AND METHODS

### 2.1 Patients

We retrospectively reviewed the medical records of consecutive patients who underwent LUR or RALUR for primary VUR at the Nagoya City University Hospital between March 2012 and July 2020. We included patients aged >15 years in the adult patient group and excluded those with ectopic ureters, ureteroceles and neuropathic bladders. The ureteral diameter ratio (UDR) was calculated from the measurement of the preoperative voiding cystourethrogram (VCUG) of the distal ureter, which was normalized at the L1–3 vertebral distance^13^ by one of the authors (K.M.). Renal scarring was evaluated by perioperative dimercaptosuccinic acid scintigraphy.

### 2.2 Study design

First, we reviewed the post‐operative outcomes of the paediatric (*n* = 100) and adult (*n* = 15) patient groups. Second, in the paediatric patient group, we compared the surgical findings and outcomes between the LUR and RALUR groups. Third, to elucidate the predictive factors for post‐operative VUR resolution, univariate and multivariate analyses were performed in the paediatric patient group.

### 2.3 Surgical procedures and post‐operative follow‐up

During patient selection, regarding the use of LUR or RALUR, we informed the patients or their parents that we could perform either procedure; the procedure was only selected after obtaining their consent. Because neither procedure was covered by public health insurance in Japan at the time, these surgeries were performed as clinical studies with the approval of the Institutional Review Board (nos. 44‐07‐0003, 46‐12‐0003 and 60‐22‐0032). Surgical indications included breakthrough urinary tract infection (UTI), high‐grade reflux and marked renal scarring.

LUR and RALUR were performed as described in previous studies.^7^, ^14^ Briefly, after cystoscopy and ureteral stenting in all cases, LUR was performed using three trocars (5 mm), and RALUR was performed using three robotic trocars (8 or 12 mm) with supporting ports (5 mm). In RALUR cases, the da Vinci S or Xi surgical system (Intuitive Surgical, Sunnyvale, CA, USA) was used. After the affected ureters were mobilized, ureteral diameter was measured using a paper ruler. Detrusorrhaphy was performed in a cephalad direction at 45–60° for a length of at least five times the ureteral diameter.^15^ The length was measured using a ruler in an empty bladder in every case. Subsequently, we incised the bladder muscularis to that length and embedded the ureter. Owing to the mobility of the ureter in some cases, the submucosal tunnel length becomes shorter to prevent kinking of the ureter after suturing the bladder muscularis. Before bladder muscularis suturing, the ureteral advancement technique^14^ was performed in all but a few patients. The representative video of RALUR is provided as Video S1.

Ureteral and urethral catheters are generally removed on post‐operative days 1–3. The surgical findings are shown in Tables 1 and 2. We also observed the hydrodistension (HD) grade of the ureteral orifice with VUR on cystoscopy before LUR or RALUR in every case according to the HD classification.^16^ Post‐operative complications were assessed using the Clavien–Dindo classification system.^17^ Successful outcomes were defined as radiographic resolution of VUR according to VCUG at 3–4 post‐operative months. All patients underwent urinalysis and abdominal ultrasonography every 6–12 months after VUR resolution.

**TABLE 1:** Patients' characteristics and surgical outcomes in paediatric and adult patients who underwent LUR or RALUR for primary VUR.

	Paediatric (*n* = 100)	Adult (*n* = 15)
Patients' demographics		
Male, *n* (%)	67 (67.0)	2 (13.3)
Female, *n* (%)	33 (33.0)	13 (86.7)
Age at operation, months (IQR)	28 (20–84)	279 (203–380)
Height, cm (IQR)	88.5 (81.6–120.1)	159.0 (155.3–162.2)
Weight, kg (IQR)	13.3 (11.3–22.5)	49.7 (43.2–60.5)
BMI, kg/m^2^ (IQR)	16.5 (15.4–18.0)	19.1 (17.9–24.8)
Laterality		
Unilateral, *n* (%)	38 (38.0)	8 (53.3)
Bilateral, *n* (%)	62 (62.0)	7 (46.7)
Clinical findings		
Preoperative CAP, *n* (%)	81 (81.0)	6 (40.0)
Preoperative fUTI, times	2.0 (1.0–3.0)	3.0 (1.0–4.5)
Breakthrough UTI, *n* (%)	23 (23.0)	2 (13.3)
BBD, *n* (%)	1 (1.0)	1 (6.7)
Completed toilet training, *n* (%)	39 (39.0)	15 (100.0)
Radiological findings		
Preoperative VUR grade, renal unit (%)		
I	10 (6.2)	0 (0.0)
II	10 (6.2)	5 (20.8)
III	43 (26.7)	12 (50.0)
IV	83 (51.6)	6 (25.0)
V	15 (9.3)	1 (4.2)
Duplex, *n* (%)	5 (5.0)	3 (20.0)
Paraureteral diverticulem, *n* (%)	5 (5.0)	1 (6.7)
UDR, ratio (IQR)	0.267 (0.179–0.368)	0.170 (0.114–0.235)
Renal scarring, *n* (%)	57 (57.0)	11 (73.3)
Surgical procedures		
LUR, *n* (%)	66 (66.0)	10 (66.7)
RALUR, *n* (%)	34 (34.0)	5 (33.3)
Total operative time, min (IQR)	234.5 (203.0–264.8)	264.0 (221.0–298.0)
Pneumoperitoneum/console time, min (IQR)	181.5 (147.5–214.0)	201.0 (178.0–246.0)
Ureteral dissection time, min (IQR)	18.0 (13.0–24.0)	44.5 (31.0–61.5)
Anastomosis time, min (IQR)	34.0 (30.0–42.0)	44.0 (35.0–48.0)
Blood loss, mL (IQR)	2 (1–4)	2 (1–51)
HD classification, ureter (%)		
H0	49/151 (32.5)	1/18 (5.6)
H1	72/151 (47.7)	10/18 (55.6)
H2	24/151 (15.9)	3/18 (16.7)
H3	6/151 (4.0)	4/18 (22.2)
Ureter diameter, mm (IQR)	4.0 (5.0–6.0)	6.0 (5.0–6.5)
Submucosal tunnel length, mm (IQR)	30.0 (25.0–32.0)	30.0 (30.0–30.5)
Ureteral advancement, renal unit (%)	157/162 (96.9)	16/22 (72.7)
Post‐operative outcomes		
Success rate, renal unit (%)	139/156 (89.1)	14/20 (70.0)
Complications, *n* (%)		
C‐D I	19 (19.0)	4 (26.7)
C‐D II	3 (3.0)	1 (6.7)
C‐D III	0 (0.0)	1 (6.7)
C‐D IV‐V	0 (0.0)	0 (0.0)
Post‐operative urinary retention, *n* (%)	5 (5.0)	1 (6.7)
De novo VUR, renal unit (%)	9/162 (5.6)	0/22 (0.0)
Post‐operative fUTI, *n* (%)	3 (3.0)	2 (13.3)
Follow‐up, months (IQR)	74.0 (54.0–103.0)	30.0 (24.3–57.3)

**TABLE 2:** Patients' characteristics and surgical outcomes in paediatric who underwent LUR or RALUR for primary VUR.

	LUR (*n* = 66)	RALUR (*n* = 34)	*p* value
Patients' demographics			
Male, *n* (%)	47 (71.2)	20 (58.8)	0.263
Female, *n* (%)	19 (28.8)	14 (41.2)	
Age at operation, months (IQR)	27.5 (20.0–64.0)	35.0 (20.8–102.0)	0.285
Height, cm (IQR)	88.1 (80.9–107.7)	92.5 (83.6–129.3)	0.247
Weight, kg (IQR)	12.4 (11.2–17.7)	14.5 (11.3–25.0)	0.223
BMI, kg/m^2^ (IQR)	16.5 (15.3–18.2)	16.6 (15.5–17.9)	0.827
Laterality			
Unilateral, *n* (%)	27 (40.9)	11 (32.4)	0.549
Bilateral, *n* (%)	39 (59.1)	23 (67.6)	
Clinical findings			
Preoperative CAP, *n* (%)	54 (81.8)	27 (79.4)	0.792
Preoperative fUTI, times	2 (1–3)	2 (2–3)	0.829
Breakthrough UTI, *n* (%)	18 (27.3)	5 (14.7)	0.212
BBD, *n* (%)	0 (0.0)	1 (2.9)	0.340
Completed toilet training, *n* (%)	23 (34.8)	16 (47.1)	0.282
Radiological findings			
Preoperative VUR grade, renal unit (%)			
I	5 (4.8)	5 (8.8)	0.327
II	9 (8.7)	1 (1.8)	0.099
III	22 (21.2)	21 (36.8)	**0.040**
IV	62 (59.6)	21 (36.8)	**0.008**
V	6 (5.8)	9 (15.8)	**0.048**
Duplex, *n* (%)	5 (7.6)	0 (0.0)	0.163
Paraureteral diverticulem, *n* (%)	4 (6.1)	1 (2.9)	0.659
UDR, ratio (IQR)	0.292 (0.198–0.373)	0.212 (0.162–0.328)	0.055
Renal scarring, *n* (%)	40 (60.6)	17 (50.0)	0.394
Surgical findings			
Operative time, min (IQR)			
Unilateral	192.0 (173.5–216.0)	174.0 (157.0–222.5)	0.704
Bilateral	260.0 (243.0–284.0)	235.0 (213.5–250.5)	**0.006**
Pneumoperitoneum/console time, min (IQR)			
Unilateral	152.0 (131.5–173.0)	110.0 (105.0–144.5)	**0.008**
Bilateral	216.0 (204.5–241.0)	172.0 (152.5–182.5)	**<0.001**
Ureteral dissection time, min (IQR)	17.0 (14.0–24.0)	18.0 (12.3–28.0)	0.732
Anastomosis time, min (IQR)	38.0 (32.0–45.0)	32.0 (26.3–35.8)	**<0.001**
Blood loss, mL (IQR)	1 (1–6)	2 (1–4)	0.067
HD classification, ureter (%)			
H0	29 (28.4)	20 (40.8)	**0.017**
H1	47 (46.1)	25 (51.0)	
H2	20 (19.6)	4 (8.2)	
H3	6 (5.9)	0 (0.0)	
Ureteral diameter, mm	5.0 (4.0–6.0)	4.0 (4.0–5.0)	**0.028**
Submucosal tunnel length, mm	30 (25–34)	25 (22–30)	**<0.001**
Ureteral advancement, renal unit (%)	100 (95.2)	57 (100.0)	0.163
Post‐operative outcomes			
Success rate, renal unit (%)	92 (91.1)	47 (85.5)	0.293
Complications, *n* (%)			
C‐D I	11 (16.7)	8 (23.5)	0.429
C‐D II	1 (1.5)	2 (5.9)	0.266
C‐D III	0 (0.0)	0 (0.0)	—
C‐D IV‐V	0 (0.0)	0 (0.0)	—
Post‐operative urinary retention, *n* (%)	3 (4.5)	2 (5.9)	0.771
De novo VUR, renal unit (%)	7 (6.7)	2 (3.5)	0.495
Post‐operative fUTI, *n* (%)	3 (4.5)	0 (0.0)	0.298
Follow‐up, months	68.0 (48.0–111.5)	78.0 (66.5–102.8)	0.238

### 2.4 Statistical analysis

All data were analysed using SPSS Statistics (version 24.0; IBM Japan Ltd. Tokyo, Japan). The Shapiro–Wilk test for normality was used; all continuous values are expressed as medians with IQR. To compare the surgical outcomes of LUR and RALUR, the Mann–Whitney *U* test was used for statistical analyses of continuous variables with non‐normal distributions, and Fisher's exact test was used for nominal variables. Univariate and multivariate analyses using a generalized linear mixed model were used to assess patient and procedural characteristics contributing to surgical success. In addition, receiver operating characteristic analysis was used to evaluate the cut‐off values of the predictive factors, sensitivity and specificity. A *p* value of <0.05 was considered statistically significant.

## 3 RESULTS

### 3.1 Overview of surgical outcomes of paediatric and adult patients

In total, 115 patients and 186 renal units were included in this study. Owing to the small number of adult cases, statistical comparisons with paediatric patients were not conducted; however, the demographic characteristics and surgical outcomes for all patients are shown in Table 1.

In the paediatric patient group, male patients and bilateral cases were predominant. Median age at operation was 28 months, continuous antibiotic prophylaxis (CAP) was administered in 81.0% of cases, and toilet training was completed in 39.0% of cases. LUR was more commonly performed, and the overall SR was 89.1%. There were no cases where post‐operative complications above Clavien–Dindo grade III occurred; five cases experienced transient urinary retention, which improved in a few days of urethral catheterization. The median post‐operative follow‐up period was 74.0 months, and 3 cases developed febrile UTI during this period.

Among the adult patients, female patients were predominant, and the median age at operation was 23 years and 3 months (279.0 months). CAP was administered in only 40% of cases, and 70% had VUR of grade III or lower, indicating that many cases were relatively mild. Several cases required a prolonged ureteral dissection, with a median time of 44.5 min. In some cases, ureteral advancement suture could not be completed owing to limited ureteral mobility and a restricted operative field around ureterovesical junction caused by insufficient dissection. The overall SR was 70.0%, and febrile UTI occurred in two cases during mean post‐operative follow‐up periods of 30.0 months. There were no cases of repeated VCUG after the first assessment among both paediatric and adult patients. We encountered a case of compartment syndrome in the left lower leg of a 16‐year‐old boy immediately after experiencing RALUR. An immediate relaxing incision was made, and the patient recovered fully without leg paralysis.

### 3.2 Comparison of LUR and RALUR among paediatric patients

No significant differences in patient demographics were observed between the LUR and RALUR groups in paediatric patients. However, the proportion of patients with grade V disease was significantly higher in the RALUR group than that in the LUR group (*p* = 0.048) (Table 2). The operative time for bilateral cases, pneumoperitoneum and console time were significantly shorter in the RALUR group than in the LUR group. Although the ureteral dissection time was not significantly different, the anastomosis time was significantly shorter in the RALUR group than in the LUR group (*p* < 0.001). HD classification grades and ureteral diameters were significantly higher in the LUR group than in the RALUR group. Although the submucosal tunnel length was significantly longer in the LUR group than in the RALUR group, the overall SR was not significantly different between the two groups (91.1% and 85.5%, respectively). No significant differences in post‐operative complications, including acute urinary retention and febrile UTI, were observed.

### 3.3 Factors associated with VUR resolution in paediatric patients

**FIGURE 1:**
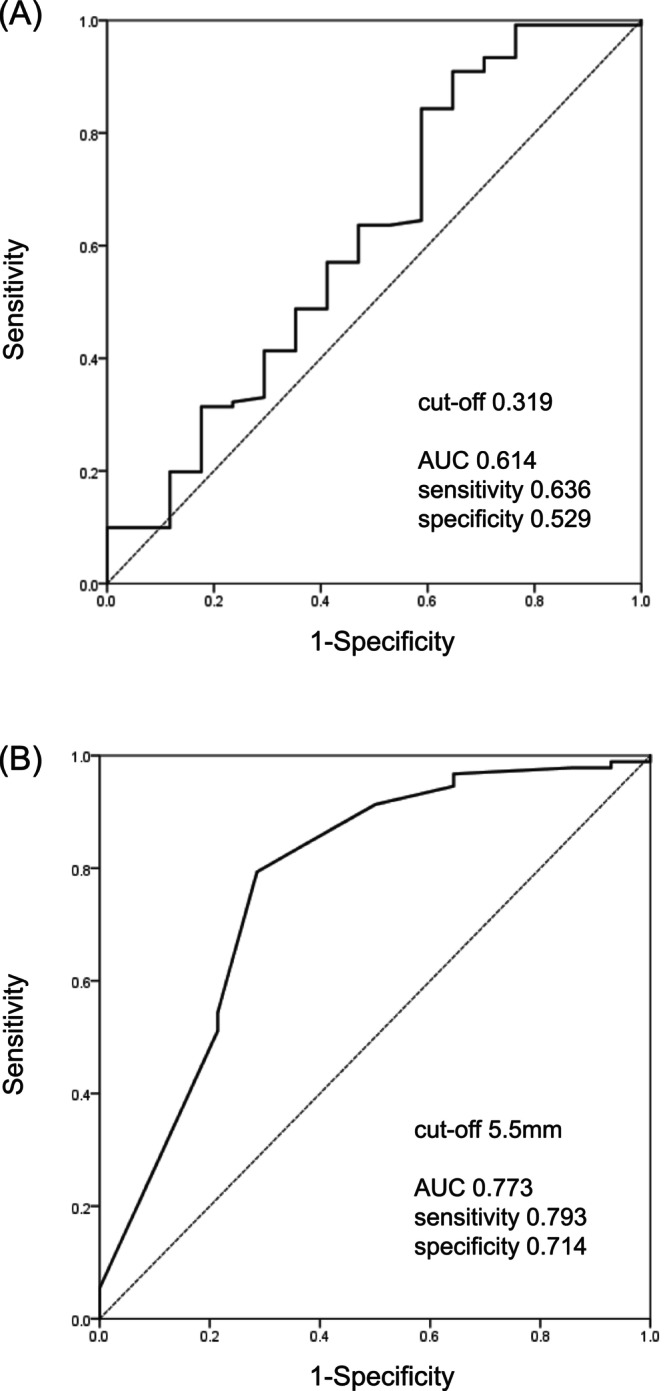
ROC curves derived from paediatric patients depicting (A) UDR < 0.319 and (B) ureteral diameter <5.5 mm to predict VUR resolution after LUR or RALUR. LUR, laparoscopic ureteral reimplantation; RALUR, robot‐assisted laparoscopic ureteral reimplantation; ROC, receiver operating characteristic; UDR, ureteral diameter ratio; VUR, vesicoureteral reflux.

Univariate analysis revealed that UDR (odds ratio [OR], 0.678; *p* = 0.043) and ureteral diameter (OR, 0.683; *p* = 0.015) were significant predictive factors for VUR resolution in paediatric patients. However, the VUR grade, surgical procedure and submucosal tunnel length were not significantly associated (Table 3). In the multivariate analysis, only ureteral diameter (OR, 0.721; *p* = 0.046) was an independent risk factor for VUR resolution in paediatric patients (Table 3). Furthermore, analysis of the receiver operating characteristic curve indicated that the optimal cut‐off values of UDR and ureteral diameter for predicting VUR resolution were 0.319 and 5.5 mm, respectively. The area under the curve values (95% confidence intervals) of the UDR and ureteral diameter were 0.614 (0.455–0.773) and 0.773 (0.622–0.924), respectively. The sensitivity and specificity of UDR were 63.6% and 52.9%, respectively. The sensitivity and specificity of ureteral diameter were 79.3% and 71.4%, respectively (Figure 1).

**TABLE 3:** Contributing factors associated with VUR resolution.

	Univariate analysis		Multivariate analysis	
Variable	OR (95% CI)	*p* value	OR (95% CI)	*p* value
Female	1.487 (0.364–6.064)	0.578		
BMI (per 1)	1.061 (0.792–1.422)	0.688		
VUR grade (per 1 grade)	0.997 (0.567–1.751)	0.991		
bUTI (ref = no)	2.228 (0.410–12.696)	0.344		
Renal scarring (ref = no)	0.621 (0.191–2.017)	0.425		
UDR (per 0.1)	0.678 (0.466–0.988)	**0.043**	0.692 (0.468–1.024)	0.066
Surgical procedures (ref = RALUR)	1.785 (0.509–6.261)	0.363		
Operative time (per 10 min)	0.884 (0.775–1.008)	0.066		
HD classification; H1 (ref = H0)	0.544 (0.113–2.633)	0.447		
HD classification; H2 (ref = H0)	0.513 (0.075–3.533)	0.495		
HD classification; H3 (ref = H0)	0.258 (0.012–5.665)	0.387		
Ureteral diameter (per 1 mm)	0.683 (0.502–0.928)	**0.015**	0.721 (0.523–0.994)	**0.046**
Submucosal tunnel length (per 1 mm)	0.908 (0.821–1.004)	0.059	0.935 (0.837–1.044)	0.231
Ratio (tunnel/ureteral diameter) (ref = <5)	5.579 (0.898–34.664)	0.065	5.068 (0.779–32.981)	0.089

## 4 DISCUSSION

Reports on the surgical outcomes of adult patients with VUR are few because most patients receive treatment during childhood or the reflux spontaneously resolves in several cases. In the present study, we reviewed the surgical outcomes in 15 adult patients who underwent LUR or RALUR. Only two cases were experienced in post‐operative febrile UTI, and no case needed secondary procedures during the follow‐up period. However, radiological SR was relatively lower herein (70.0%) compared to a previous study.^18^ Contrary to that observed in paediatric patients, ureteral dissection in adults required a relatively longer time, with median duration of 44.5 min, and tended to have a lower rate in achieving ureteral advancement suture. This could be attributed to the deep pelvis and large amount of fatty tissue surrounding the ureter in adult patients, making identification of the ureter difficult and time consuming.^18^

Chandrasekharam et al. performed a meta‐analysis involving paediatric patients and reported that RALUR had a longer operative time and lower SR than LUR with comparable complication rates.^19^ They also reported a high proportion of grade V and bilateral RALUR. In the present study, the pneumoperitoneum/console time was significantly shorter in the RALUR group than in the LUR group, consistent with the findings of Esposito et al.^20^ The short console time in the RALUR group could be attributed to the advantages of suturing the robotic platform and the presence of a supporting trocar. The SRs and complication rates were comparable between the LUR and RALUR groups. The median post‐operative follow‐up period was >5 years in paediatric patients. During this period, there were only three cases of febrile UTI in the LUR group, suggesting that the resolution of VUR and long‐term outcomes were favourable.

In a systematic review of RALUR, Esposito et al. reported that the overall patient SR was 92% among paediatric patients.^8^ They also reported that the results of RALUR gradually improved owing to these innovative techniques. A recent systematic review reported greater success (92.8%) and lower failure (5.2%) rates than those in a previous study (90.9% and 9.2%, respectively).^8^ Improvements in surgical outcomes have been reported even within a single institution and among the same surgeons.^21^ In the present study, the overall SR (85.5%) was comparable to that in previous reports but slightly lower than expected. This is likely attributable to the fact that more than half of the cases involved bilateral instances or VUR grades IV and V. In addition, some studies have reported that SR is defined by clinical success because of the avoidance of routine post‐operative VCUG.^22^ In this context, the clinical SRs were 95.5% and 100% for the LUR and RALUR, respectively.

Fundamentally, the pathology of VUR involves failure of the anti‐reflux mechanism of the ureterovesical junction, such as passive flap‐valve mechanisms.^23^ To correct this abnormality, the importance of intramural ureteral length, such as the ‘5:1 rule’,^24^ or the morphology of the ureteral orifice^25^ has been emphasized. In this study, we performed preoperative cystoscopy in every patient and assessed the shape of the ureteral orifice using HD classifications.^16^ Univariate and multivariate analyses showed that the HD classification did not contribute to the resolution of VUR, suggesting that intramural ureteral length is critical in MIS when using extravesical approaches. Several patient‐related factors such as laterality, age and preoperative bladder and bowel dysfunction (BBD) have been reported as risk factors for persistent VUR.^26^ In this study, no significant differences were observed in these patient‐related factors, possibly because of the low incidence of BBD and predominance of younger children. Although preoperative BBD has been reported to predict post‐operative urinary retention after RALUR,^27^ the low incidence of post‐operative urinary retention may be related to the patient characteristics.

The ‘LUAA’ technique proposed by Gundeti et al. is another contributing factor.^12^ The importance of securing a sufficient length of the submucosal tunnel or incorporating ureteral adventitia in detrusorraphy has been emphasized in a multicentre study.^28^ Ureteral advancement sutures have been deemed useful,^14^ suggesting that these procedures contribute to surgical success. In this study, we identified the ureteral diameter as a contributing factor to surgical success. We intraoperatively measured the ureteral diameter immediately after exposure and attempted to create a submucosal tunnel at least five times. The present finding on such a distinct numerical factor is novel. Despite the limitation of the ureteral diameter being measurable only intraoperatively, UDR, which can be calculated via preoperative VCUG, has proven useful. For some high‐grade VUR cases with orthotopically dilated ureters, ureteral remodelling such as tapering or tailoring may be necessary. However, recent reports have revealed that ureteral tapering and tailoring are unnecessary to achieve reflux resolution in grades III–V VUR when using both open surgery and RALUR.^10^ Robotic surgery allows for a greater range of complex manoeuvres than laparoscopy. Thus, the ability to perform intricate procedures such as LUAA may lead to improved treatment outcomes for RALUR.

The present study has some limitations. We combined the data of the LUR and RALUR groups for comparison between paediatric and adult patients, because separating the surgical techniques would have resulted in a small number of cases for analysis. Nevertheless, among paediatric patients, we compared the surgical outcomes between the LUR and RALUR groups. Caution is required when interpreting the surgical outcomes, as the patient backgrounds differ, including variations in the number of trocars and VUR grade. Furthermore, selection bias exists in this study owing to the lack of randomization in the selection of procedures (LUR or RALUR). Additionally, the present study was conducted at a single institution with a single surgeon performing the procedures; studies including large case numbers or multiple institutions are needed to validate our results. Using an extravesical approach, we identified the UDR as a contributing factor to the resolution of VUR after MIS. The UDR is a relatively recent objective measurement that appears to be a new tool for predicting clinical outcomes and success after endoscopic injection for VUR.^29^ However, there was considerable variability in UDR measurements among the evaluators.^30^ Therefore, analyses involving multiple evaluators and development of objective indicators using imaging technology are required.

## 5 CONCLUSIONS

We retrospectively assessed the surgical outcomes of paediatric and adult patients who underwent LUR or RALUR and elucidated the factors contributing to the resolution of VUR. The overall SR was higher in the paediatric group than in the adult group. Among paediatric patients, the overall SR and complication rate were not significantly different between the procedures. Both LUR and RALUR are favourable and safe procedures for paediatric patients with primary VUR. Furthermore, the ureteral diameter measured during surgery was significantly associated with VUR resolution regardless of whether the robotic platform was used or not. UDR was also found to be a useful preoperative tool for predicting SR. To improve SR after LUR or RALUR, a sufficient length of the submucosal tunnel must be ensured based on intraoperative ureteral diameter measurements.

## ETHICS COMMITTEE APPROVAL

This study was approved by the ethics committee of Nagoya City University (NCU no. 60‐22‐0032). LUR and RALUR were performed at our hospital with the approval of the ethics committee of Nagoya City University (NCU no. 44‐07‐0003 (UMIN000021212) and NCU no. 46‐12‐0003 (UMIN000021211), respectively).

## AUTHOR CONTRIBUTIONS

**Kentaro Mizuno**: Conception and design of this study, data acquisition, data analysis and interpretation and writing of the original draft. **Hidenori Nishio**: Data collection, review and editing of the original draft. **Daisuke Matsumoto**: Review and editing of the original draft. **Takuya Sakata**: Review and editing of the original draft. **Akihiro Nakane:** Review and editing of the original draft. **Hideyuki Kamisawa**: Review and editing of the original draft. **Satoshi Kurokawa**: Review and editing of the original draft. **Tetsuji Maruyama**: Review and editing of the original draft. **Keiichi Tozawa**: Supervision. **Takahiro Yasui**: Supervision. **Yutaro Hayashi**: Supervision, review and editing of the original draft.

## CONFLICT OF INTEREST STATEMENT

The authors declare no conflict of interest.

## Supporting information


**Video S1.** The representative video of right RALUR (1 year and 11 months old, male, grade IV) (2 min 6 sec, 26 MB). RALUR, robot‐assisted laparoscopic ureteral reimplantation.

## Data Availability

The datasets generated and/or analysed during the current study are not publicly available but are available from the corresponding author upon reasonable request.
